# Immunomodulatory Functions of Mesenchymal Stem Cells in Tissue Engineering

**DOI:** 10.1155/2019/9671206

**Published:** 2019-01-13

**Authors:** Haojiang Li, Shi Shen, Haitao Fu, Zhenyong Wang, Xu Li, Xiang Sui, Mei Yuan, Shuyun Liu, Guiqin Wang, Quanyi Guo

**Affiliations:** ^1^Institute of Orthopedics, Chinese PLA General Hospital, Beijing Key Lab of Regenerative Medicine in Orthopedics, Key Laboratory of Musculoskeletal Trauma War Injuries, PLA, No. 28 Fuxing Road, Haidian District, Beijing 100853, China; ^2^Department of Microbiology and Immunology, Shanxi Medical University, Taiyuan, China; ^3^Department of Bone and Joint Surgery, The Affiliated Hospital of Southwest Medical University, No. 25 Taiping Road, Luzhou 646000, China; ^4^School of Medicine, Nankai University, Tianjin 300071, China; ^5^First Department of Orthopedics, First Affiliated Hospital of Jiamusi University, No. 348 Dexiang Road, Xiangyang District, Jiamusi 154002, China

## Abstract

The inflammatory response to chronic injury affects tissue regeneration and has become an important factor influencing the prognosis of patients. In previous stem cell treatments, it was revealed that stem cells not only have the ability for direct differentiation or regeneration in chronic tissue damage but also have a regulatory effect on the immune microenvironment. Stem cells can regulate the immune microenvironment during tissue repair and provide a good “soil” for tissue regeneration. In the current study, the regulation of immune cells by mesenchymal stem cells (MSCs) in the local tissue microenvironment and the tissue damage repair mechanisms are revealed. The application of the concepts of “seed” and “soil” has opened up new research avenues for regenerative medicine. Tissue engineering (TE) technology has been used in multiple tissues and organs using its biomimetic and cellular cell abilities, and scaffolds are now seen as an important part of building seed cell microenvironments. The effect of tissue engineering techniques on stem cell immune regulation is related to the shape and structure of the scaffold, the preinflammatory microenvironment constructed by the implanted scaffold, and the material selection of the scaffold. In the application of scaffold, stem cell technology has important applications in cartilage, bone, heart, and liver and other research fields. In this review, we separately explore the mechanism of MSCs in different tissue and organs through immunoregulation for tissue regeneration and MSC combined with 3D scaffolds to promote MSC immunoregulation to repair damaged tissues.

## 1. Introduction

The combination of MSCs and TE can promote the immunoregulatory properties of MSCs than MSCs alone can. MSCs can regulate immune responses, especially adaptive immune response. The addition of tissue engineering techniques can affect this role of MSCs and is closely related to the material and shape of the cell carrier scaffolds. Through the introduction of the immunomodulatory capacity of MSCs and the application of tissue engineering scaffolds, the paper discusses the mechanism of MSC immune regulation in different organs (cartilage, bone, cardiovascular, and liver) and the effect of TE on the immune regulation of MSCs.

### 1.1. Immune Regulation of Mesenchymal Stem Cells in the Microenvironment

The interaction between mesenchymal stem cells (MSCs) and immune cells is complex. MSCs can regulate immune cells through cell contact and secretion and can directly act on immune cells to inhibit their activity. Cells that express immunosuppressive properties on the cell surface, such as programmed death-ligand 1 (PD-L1) and Fas ligand (Fas-L) [1, 2], bind to receptors on the surface of immune cells, resulting in immune cell loss of function. Evidence has suggested that MSCs bind to activated immune cells, which may keep them in close proximity and thus enhance immunosuppressive effects [3]. In addition to their direct action on immune cells, MSCs can also inhibit immune cells by secreting cytokines, including transforming growth factor-*β* (TGF-*β*), hepatocyte growth factor (HGF), and prostaglandin E2 (PGE2), as well as other anti-inflammatory factors [4, 5]. For example, MSCs secrete TGF-*β* and other factors, which can promote the induction of regulatory T cells (Tregs) [6] and macrophages [7], and in this way transmit their immunosuppressive effects to other cells to activate different immunosuppressive mechanisms. MSCs express TNF-*α*-stimulated gene/protein 6 (TSG-6) which mediates the regulation of immune inflammation. It antagonizes the binding of CXCL8 to heparin by interacting with the GAG-binding site of CXCL8, thereby inhibiting CXCL8-mediated neutrophil chemotaxis [8]. Among them, TSG-6 can inhibit extravasation of leukocytes (mainly neutrophils and macrophages) at the site of inflammation [9]. TSG-6 is another key factor that plays an important role in tissue repair function in human MSCs and is demonstrated in mouse models of myocardial infarction, peritonitis, and acute corneal and lung injury [10–13].

Therefore, MSCs play a central role in maintaining immune homeostasis by interacting with cytokines, chemokines, and cell surface molecules. Previous studies on the immune regulation of MSCs have focused on the interaction between MSCs and B lymphocytes, natural killer cells, and dendritic cells. More recently, the use of MSCs in the repair of tissue damage and regulation of the inflammatory response has attracted increasing attention with respect to macrophage and T cell regulation.

MSCs have significant immunomodulatory capacity and play a role in both the innate and adaptive immune systems. In recent years, research has focused on the repair of tissue damage by stem cells, and there has been a great deal of interest in understanding the role of MSCs in the adaptive immune response. MSCs negatively regulate the activation and proliferation of T cells (including CD4^+^ and CD8^+^ cells) by cell contact and the secretion of inflammatory soluble factors [14]. The data indicate that MSCs inhibit proliferation by inducing G0 arrest in the T cell cycle [15, 16]. MSCs can also induce T cell apoptosis mediated by the Fas-L-dependent pathway [17]. Under normal conditions, MSCs can promote the survival of T lymphocytes [18] and stimulate their proliferation through interleukin- 6 (IL-6) dependent pathways [19]. However, with activation of the immune system following tissue damage, T cell-derived interferon- *γ* (IFN-*γ*) activates the immunoregulatory properties of MSCs, resulting in suppression of the activation and proliferation of immune cells [20]. Then, MSCs upregulate the expression of indamine 2 (IDO), HGF, PD-L1, PGE2, and cyclooxygenase-2 to regulate immune function [21, 22]. Experiments have shown that more than 30 soluble factors are involved in the immune regulation of MSCs during the activation and proliferation of T lymphocytes [23], including HGF, TGF-*β* [4], IDO [24], PGE2 [5, 25], nitric oxide (NO) [26], and IL-10 [25]. It was also found that adenosine produced by MSCs reduces T cell proliferation by binding to adenosine receptors on the surface of lymphocytes [27, 28]. The ability of MSCs to inhibit T cell activation and alter T cell polarization remains a major focus of many MSC immunomodulatory studies, and soluble signals and pathways that control the interaction between MSCs and T cells are compared to other leukocyte populations. However, the immune microenvironment composed of inflammatory cytokines plays a key role in stimulating the innate and adaptive immunomodulatory activities of MSCs. Inhibition of T cell proliferation and activation by MSCs was induced by the IFN-*γ* induced expression of indoleamine 2,3-dioxygenase (IDO). Although pretreatment with IFN-*γ* is commonly used for direct MSC immunomodulatory activity prior to transplantation, transient effects resulting from pretreatment may limit the regulation of immune response by MSCs. The addition of tissue engineering technology can precisely improve and continuously induce the immunomodulatory activity of MSC to a certain extent. In order to overcome these difficulties, local transplantation of MSCs aggregates can improve the local inflammatory environment of the cells at the injection site, while increasing the expression of immunoregulatory factors. The authors believe that MSCs can maintain the structural basis of cell-cell and cell-matrix contact by means of aggregate delivery, which can prevent cell loss due to apoptosis and better implant into host tissues [29]. In one experiment, it was found that by constructing mesenchymal stem cells in a three-dimensional state, the immunosuppressive effect of T cells can be enhanced by continuously presenting bioactive IFN-*γ*, compared with MSCs pretreated alone. Microparticle delivery of IFN-*γ* in MSC spheroids can maintain immunomodulatory activity [30]. Found in a study on bone regeneration, three-dimensional cultured clumps of a mesenchymal stem cell (MSC)/extracellular matrix (ECM) complex (C-MSC) consists of cells and self-produced ECM. C-MSCs can use ECM as a cell scaffold to regulate in vitro cell function and induce successful bone regeneration. IFN-*γ* pretreatment effectively enhanced the immunomodulatory capacity of C-MSCs. X-transplantation of C-MSC*γ* into the skull of immunocompetent mice induced bone regeneration, while C-MSC xenograft failed and induced T cell infiltration [31]. In addition to regulating secretion by MSCs, T cells can exert a similar effect through cell contact. The attraction of MSCs to T cells has been explained by the expression of high levels of the leukocyte chemokine ligands CXCL9, CXCL10, and CXCL11. Neutralization with CXCR3, a T cell chemokine, as well as CXCL9, CXCL10, and CXCL11 receptors, initiates the immunosuppressive effects of MSCs, revealing the role of chemokines in regulation of the stem cell-mediated immune environment [32]. In addition to CXCR3, other molecules, such as vascular cell adhesion molecule-1 (VCAM-1), intercellular adhesion molecule-1 (ICAM-1), and programmed cell death protein 1 (PD-1), are also involved in contact inhibition of T cells by MSCs [1, 33, 34]. The effective immunosuppression of mesenchymal stem cells (MSCs) is caused by IFN-*γ* and is accompanied by the simultaneous presence of three other proinflammatory cytokines, TNF-*α*, IL-1a, or IL-1b. These cytokine combinations stimulate several chemokines and inducible nitric oxide synthase (iNOS) expressed by MSCs. Chemokines cause T cells to migrate to the vicinity of MSCs, where nitric oxide (NO) inhibits T cell immune responses [32]. Pretreatment of MSC by proinflammatory cytokines is a key link in the production of immunosuppressive properties.

Some reports have suggested that MSCs can not only reduce M1 infiltration [35] but can also reprogram macrophages from the inflammatory M1 phenotype to the anti-inflammatory M2 phenotype [36, 37]. In recent years, the effect of MSCs on macrophages has become increasingly clear. MSCs within tissues can induce macrophage migration and turn it into a regulatory phenotype [7, 37]. Coculture with MSCs can induce macrophages to increase IL-10 expression, reduce the levels of tumor necrosis factor-*α* (TNF-*α* and IL-12, and lower the expression of the costimulatory molecules CD86 and HLA class II to reduce inflammation [7, 36]. Studies have shown that MSC-mediated M2 macrophage polarization depends on the secretion of soluble factors, including PGE2, TNF-inducible gene-6 (TSG-6, IL-6, IDO, and TGF-*β*1 [12, 37, 38]. PGE2 is considered an important factor in the initiation of macrophage phenotypic changes [5].

The role of MSCs and Tregs in suppressing T cell proliferation has triggered interest in the factors and media involved. The current view is that the key factors involved in MSC induction of classical CD4^+^ CD25^+^ Foxp3^+^ Tregs are MSC-derived TGF-*β* and PGE2 [39–41]. A number of mediators and mechanisms have been proposed to participate in the role of MSCs in promoting this classical Treg phenotype. MSCs have been shown to induce Foxp3 and CD25 expression in CD4^+^ T cells by direct cell contact, followed by production of MSC-derived TGF-*β*1 and PGE2 [40, 41]. MSCs excreting TGF-*β*1 can also directly induce the production of CD4^+^ CD25^+^ Foxp3^+^ Tregs [42]. PGE2 regulates the expression of Foxp3 in human lymphocytes and induces the regulatory phenotype of CD4^+^ CD25^−^ T cells by modulating the expression of Foxp3, thus contributing to Treg function [43]. In one study, when MSCs were cocultured with allogeneic Tregs, MSCs enhanced the immunosuppressive capacity of Tregs, and this effect was accompanied by IL-10 production and upregulation of PD-1 receptors on Tregs [44].

### 1.2. Effects of Tissue Engineering Materials and Structure on the Immune Regulatory Ability of MSCs

Tissue engineering (TE) and regenerative medicine applications are aimed at improving or replacing damaged biological functions by stimulating the body's inherent regenerative capacity or by replacing damaged tissues. TE implants include biomaterials, which may be natural, synthetic, or derived from deassimilated (xenogeneic, allogeneic, or autologous) materials and/or cells derived from allogeneic or autologous sources. Given the biomaterials and antigens that exist, TE implants are usually immunostimulatory. As a foreign body, the TE implant triggers a foreign body reaction (FBR) when introduced into an organism. When the reaction occurs, monocytes are recruited to the site of implantation under signals from IL-4 and IL-1, for example, followed by differentiation into macrophages [45, 46]. By identifying surface molecules on the implanted material, macrophages phagocytose foreign bodies and form foreign body giant cells (FBGCs) [46]. The main function of FBGCs is to parcel foreign agents by secreting degradative agents (such as superoxide and free radicals) to the lesions followed by avascular collagen deposition [46]. However, there are also reports of tissue-engineered implants forming FBGCs. This process affects the rate of degradation of the scaffold and the subsequent immune response [47, 48]. Inflammatory cells have been shown to play an important role in regeneration, where the implant induces inflammation [49].

TE implant material properties are an important factor for maximizing cell recruitment and differentiation. The TE materials are selected according to the following characteristics: (i) ability to provide appropriate mechanical support to the tissue, (ii) ability to determine the digestibility of the scaffold [50], and (iii) ability to trigger the appropriate immune response to promote tissue regeneration and healing [48]. The choice of materials affects the likelihood of inflammation. Many naturally occurring biomaterials have intrinsic anti-inflammatory signals, including high molecular weight hyaluronic acid (HA) and chitosan [51], which can reduce reactive oxygen species [52, 53]. However, for most materials, the use of anti-inflammatory drugs has been more extensively studied in anti-inflammatory repair of tissue defects. In addition, with respect to the regulation of posttransplant inflammation by MSC composite scaffolds, in one study it was found that MSCs in TE can regulate macrophage activation and attenuate the FBR through continuous cross-talk with inflammatory cells [54]. Consequently, with a greater understanding of stem cell immune regulation, the use of TE materials constructed with stem cells will be increasingly important.

In addition to the choice of scaffold materials, the structure and shape of the scaffold in TE can affect inflammation. In a study on the effect of the geometry of the implanted material on its biocompatibility *in vivo*, it was found that the choice of stent had an effect on inflammation. Experimental studies have found that implanted spherical materials in various biomaterials (including hydrogels, ceramics, metals, and plastics) can significantly reduce FBRs and fibrosis depending on the diameter of the materials [55]. In a study of MSCs, construction of a three-dimensional (3D) structure in the scaffold microstructure affected the occurrence of posttransplant inflammation. Compared with conventional two-dimensional (2D) culture, 3D culture reduced macrophage recruitment and produced the anti-inflammatory proteins PGE2 and TSG-6 [56]. In recent years, the differences in MSC immunoregulation between 3D and 2D culture conditions have been studied (Table 1), including differential expression of 3D stem cell culture and conventional 2D culture in recent years. Current spheroidal culture of stem cells is most common in 3D culture [57–59], but there are also reports on polymer scaffolds [60] and 3D culture systems [61].

In the study by Emmanuel Pinteaux, MSCs increased the secretion of anti-inflammatory markers in the 3D environment, and 3D MSCs reduced the secretion of tumor necrosis factor TNF-*α* induced by LPS. These data highlight the importance of optimizing the initiation of therapeutic and culture conditions to maximize the therapeutic potential of MSC spheroids [58]. In the study by Catarina R. Almeida, macrophages in chitosan scaffolds promoted a significant increase in fibroblast recruitment rather than a significant increase in MSCs. However, macrophages that interact with MSCs in the scaffold are no longer able to recruit fibroblasts. This study demonstrates the potential of scaffolds to regulate regeneration through immune regulation [60]. In the Ren-He Xu study, the 3D construct was compared to monolayer-cultured BMSC (BMSCML). After IFN-*γ* treatment, a series of anti-inflammatory and proinflammatory genes including IDO, PD-L1, CCL2, and CXCL-10 were upregulated in the 3D group compared to untreated controls and expressed in all three IFN-*γ*-treated samples. The change of inflammatory cytokine IL-6 is small and IL-8 is decreased [59]. In a study by Sang Hun Lee, MSC spheroids showed an increase in IDO expression, as well as increased M2 macrophage ratio and reduced macrophage proliferation, compared to 2D cultured MSCs. Transplantation of MSC spheroids improved the survival rate of experimental mice and reduced the inflammatory response [57]. In the study by Christoph Giese, both TNF-*α* and IFN-*γ* were significantly inhibited in the scaffold construct. However, the production of other cytokines IL-1, IL-6, and IL-12 was also induced [61].

In many experiments, although stem cells have found to promote repair, the mechanism of repair has not been clearly explained. Does immunomodulation play a key role in this? There is no clear study on the fate and duration of stem cells during treatment and whether stem cells from different sources differ in immune regulation. In many experiments, the measurement of preinflammation factors should be used as a criterion for stimulating the immune regulation of MSCs. The effect of the material, the porosity, and the shape of the scaffold on MSCs still require further investigation. The spatial characteristics of the 3D scaffold structure also have certain limitations in culture. According to its structure, the diffusion of nutrients, oxygen, and waste through the stent is size-dependent, resulting in insufficient oxygen and nutrient supply [62–64]. In a harsh microenvironment, this can affect cell viability [64].

## 2. Effect of Stem Cell Immunomodulation on Tissue and Organ Injury Repair

### 2.1. Tissue of Regeneration

In the development of inflammation in effective tissue regeneration, the regenerative repair of tissue is a continuous process involving the interaction of stem cells with tissue-retained and recruited immune cells. The regression of the inflammatory phase and the transition to the regeneration phase are critical to the outcome of postinjury repair, which may aggravate the disease and impede repair. The application of stem cell combined tissue engineering technology has been widely used in liver, heart, and skeletal systems. In orthopedic systems, the application of stem cell tissue engineering technology in connective tissue such as cartilage and meniscus still has great development prospects.

### 2.2. Regulatory Effects of MSCs on Cartilage Macrophages

In clinical practice, cartilage injury is a multifactorial disease. At present, therapeutic interventions do not provide satisfactory therapeutic results and can lead to a decline in exercise capacity. Cartilage injury occurs primarily at the joint site, and the regenerative capacity of cartilage tissue is limited when the articular cartilage is damaged. If treatment is not effective, injury can lead to osteoarthritis (OA). Cartilage degeneration and inflammation are key features of OA, an inflammatory and degenerative joint disease that affects the entire joint and causing pain, deformity, and loss of function [65, 66]. In recent years, with the in-depth study of the immune microenvironment in tissue repair, we believe that creating a suitable microenvironment during cartilage regeneration can promote this process.

Mesoderm-derived MSCs are perivascularly derived pluripotent stem cells that have the ability to differentiate into multiple cell types, including cartilage, bone, and adipocytes [67]. In previous experiments, MSCs have been used in preclinical studies of sepsis and acute respiratory syndrome treatment, suggesting that the paracrine effect of stem cells plays a role in the regulation of inflammation [68, 69]. Moreover, MSC regulation in the immune microenvironment promotes MSC chondrogenesis [70]. The induced polarization of macrophages and exosome secretion by stem cells also play a regulatory role in the immune microenvironment [70].

**Figure 1:**
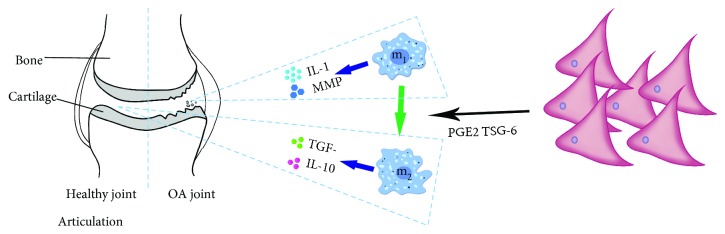
MSCs affect the development of arthritis through immunosuppression. MSCs promote the differentiation of macrophage to M1 by secreting PGE2 and TSG-6 and secrete anti-inflammation factors against soft inflammatory lesions. The blue arrow indicates the secretion of cellular cytokines, and the green arrow indicates the differentiation process.

The immunomodulatory effects of stem cells are particularly important in TE development. The pathogenesis of arthritis is partly mediated by the action of proinflammatory cytokines, such as IL-1, which are elevated in the synovial fluid of joints in OA [71–73]. IL-1 induces the release of proinflammatory cytokines, such as matrix metalloproteinases (MMPs) and NO, and downregulates the expression of primary extracellular matrix (ECM) components to promote catabolism and antisynthesis in the metabolic signaling of articular chondrocytes [72, 73]. The MSC-based engineered cartilage can promote macrophage polarization to the M2 phenotype, enabling macrophages to exhibit anti-inflammatory properties, including upregulation of CD206, increased synthesis of IL-10, reduced secretion of IL-1*β*, and expression of genes indicative of the M1 to M2 transition. It has been suggested that MSC-based TE constructs may improve scaffold-induced inflammation and cartilage tissue regeneration through M2-polarized macrophages (Figure 1). The bone marrow stromal cell (BMSC) based engineered cartilage can inhibit inflammation *in vivo* by increasing the M2 polarization of macrophages, resulting in improved survival compared with the use of chondrocytes as seed cells [70]. However, with respect to the immunosuppressive properties of MSCs, conflicting results have been reported for cartilage-differentiated cells. Ren et al. showed that chondrogenic differentiation of MSCs can increase the anti-host immune response following allogeneic transplantation [33]. However, another study highlighting the differentiation of MSCs into chondrocytes found that MSCs exhibit similar properties in terms of suppressing T cell responses in allogeneic models [74]. Therefore, the relationship between the differentiation of stem cells and the change in immunosuppressive capacity during cell transplantation is particularly important for the immune regulation of stem cells. In one study of MSC-mediated repair of cartilage injury, MSC secretion of exosomes, to promote tissue repair, also involved regulation of the immune response. Secretion of exosomes can promote the enrichment of CD163^+^ M2 cells, decrease the infiltration of CD81^+^ M1 cells, and reduce the release of related inflammatory factors [75]. From the above observations, it has been suggested that effective cartilage regeneration can be achieved by coordinated mobilization and efficient activation of multiple cell types.

In the past experiment, the immunogenicity of MSCs was not fully explored. With the changes of MSCs after implantation, the immunogenicity of MSCs will affect the occurrence of inflammatory reaction. How to maximize the low immunogenicity of foreign implanted MSCs is worthy of further experimental research.

### 2.3. Regulatory Effects of MSCs on T Lymphocytes in Bone

**Figure 2:**
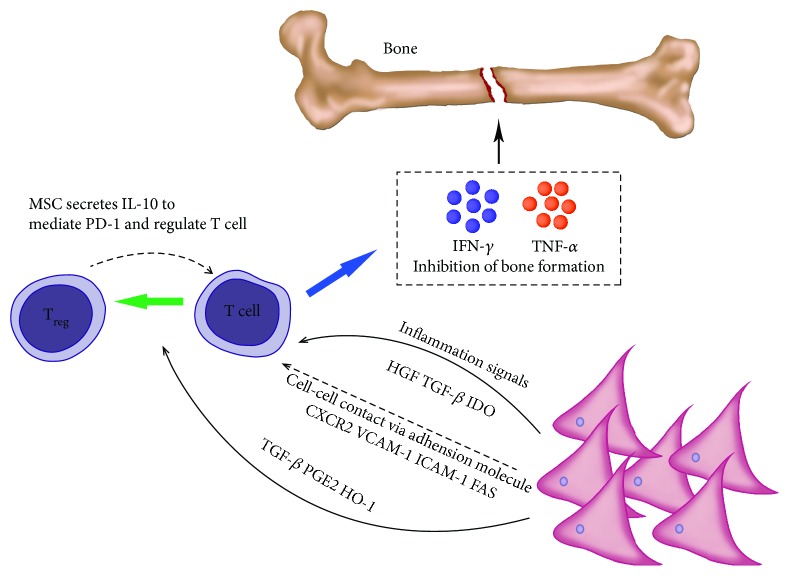
In addition to direct immunosuppressive inhibition, T cells can induce Treg cells under the regulation of MSCs and vice versa. Blue arrow: cytokine secretion, dotted arrow: inhibition of MSCs and Treg, black arrow: positive promotion, and green arrow: differentiation of T cells to Treg.

In the event of a fracture, the early inflammatory response plays a crucial role in bone healing. However, when inflammation persists, it can inhibit fracture repair. Since the innate immune system is stimulated by a variety of cytokines, it activates and reaches the site of injury after fracture [76, 77]. The immunomodulatory capacity of MSCs plays a role in both the innate and the adaptive immune response. The adaptive immune response, which is mainly composed of lymphocytes, has important implications for the fracture healing process [78, 79]. With respect to the adaptive immune response, MSCs inhibit proliferation by inducing G0 arrest in the T cell cycle [15, 16]. Moreover, MSCs can induce apoptosis of T cells mediated by the Fas-L-dependent pathway [17]. MSCs induce Foxp3 and CD25 expression in CD4^+^ T cells through direct cellular contact and secretion of TGF-*β* and PGE2 and induce classical CD4^+^ CD25^+^ Foxp3^+^ Tregs [41, 42] (Figure 2). When MSCs are cocultured with Tregs, MSCs enhance the immunosuppressive capacity of Tregs, leading to the upregulation of PD-1 receptors on Tregs via production of IL-10 [44]. MSC production of heme oxygenase- 1 (HO-1) is also involved in the induction of Tregs [80]. Reinke et al. investigated the role of T cells in MSC-mediated osteogenesis in mouse skull defects and showed that proinflammatory T cells inhibit MSC-induced bone formation by releasing IFN-*γ* and TNF-*α* [81]. Conversely, Foxp3^+^ Tregs significantly reduce TNF-*α* and IFN-*γ* levels and lead to MSC-mediated bone regeneration and skull defect repair [81]. Contrary to the view that T cells inhibit bone healing, Nam et al. reported that the proinflammatory cytokine IL-17, produced by Th17 lymphocytes, appears to mediate bone formation during fracture healing [82]. Through the regulation of immune cells, the use of MSC therapy is an attractive option for promoting bone fracture repair.

The effects of MSCs on T cells in bone damage and the effects in negative regulatory T cells were mentioned in the above experiments. However, it is still necessary to further explain the mechanism of MSCs negatively regulating T cells from the direct contact of cells and the secretion of cytokines.

### 2.4. MSCs Reduce Liver Fibrosis by Regulating Macrophage Differentiation during Liver Regeneration

**Figure 3:**
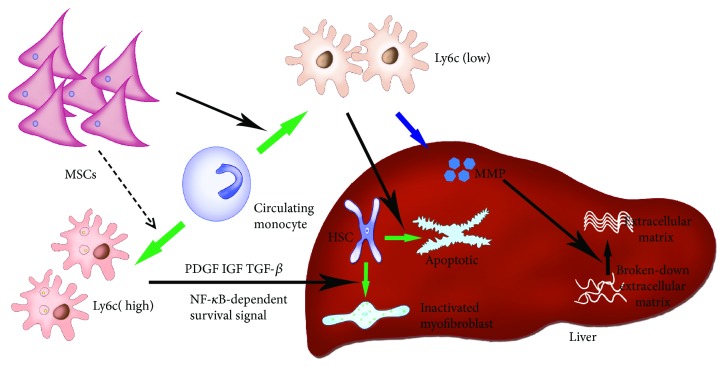
MSCs regulate the differentiation of monocytes and differentiated low-expression Ly6c macrophages in liver fibrosis through the apoptosis of HSC and the secretion of MMP against the inflammatory fibrosis of the liver. Blue arrow: cells secrete cytokines. Green arrow: differentiation and alteration of monocytes and HSC. Dotted arrow: MSCs inhibit differentiation to high expression of Ly6c macrophage. Black arrow: positive promotion of cells and cytokines.

The liver is a highly regenerative organ with a strong ability to self-regenerate. However, in chronic injury, the structure of normal hepatic lobules is destroyed or lost and is replaced by pseudo-lobules, which eventually result in regenerative failure. However, when acute or repetitive injury is caused by a toxin or viral infection, the liver can be effectively regenerated [83, 84]. This process benefits from the activation of immune cells immediately following injury, which can mobilize liver growth factors as well as initiate synergistic responses of immune cells [85, 86]. The treatment of MSCs in acute and chronic hepatic failure mainly improves immune function following hepatic injury through immunoregulatory factors released by MSCs [87, 88]. Regulation of the immune system may be a viable alternative in the treatment of liver failure. Fibrosis reflects a pathological change in liver failure. During the study of liver fibrosis, macrophages were found to perform dual functions in this process. Kupffer cells and infiltrating mononuclear cells in scar tissue following liver injury induced and activated hepatic fibroblasts to participate in the occurrence and development of hepatic fibrosis [89]. However, different subpopulations of monocytes/macrophages exhibit antifibrotic properties because of their anti-inflammatory properties [90]. Animal studies have demonstrated that the expression of Ly-6C and Gr1 on macrophages provides a better indication of their role in fibrosis. Macrophages with high expression of Ly-6C or Gr1 are profibrotic [91, 92] and are a major source of TGF-*β*, platelet-derived growth factor, and insulin growth factor-1 (IGF-1), which is used to activate HSCs and initiate NF-*κ*B-mediated fibroblast survival signals [93]. By contrast, low-Ly-6C-expressing macrophages [94, 95] exhibit antifibrotic properties [94, 96, 97]. They produce MMPs that directly degrade the ECM [94] and promote hepatic stellate cell (HSC) apoptosis through caspase-9 and TNF-related apoptosis-inducing ligand-dependent mechanisms. This duality of macrophage function has been demonstrated in a series of fibrotic mouse models (CCl_4_, dimethylnitrosamine, and thioacetamide models) [97–99]. Mononuclear cells cause regression of fibrosis due to matrix degradation [94, 95] (Figure 3). In another study, different subpopulations of monocytes and differentiated macrophages were shown to exhibit different effects on hepatic fibrosis [100]. Thus, it has been suggested that the immunomodulatory capacity of MSCs will be important in the treatment of hepatic fibrosis. In liver injury, MSCs mediate the antifibrotic effect of regulation of the conversion of macrophages to the anti-inflammatory M2 type, which is important in the treatment of hepatic fibrosis.

In the control of inflammatory response and correction of liver fibrosis in MSCs, the mechanism of action of MSCs against liver fibrosis is lacking, thus providing more insights for optimal treatment.

### 2.5. MSCs Regulate Angiogenesis through Regulation of Macrophages in the Damaged Myocardium

**Figure 4:**
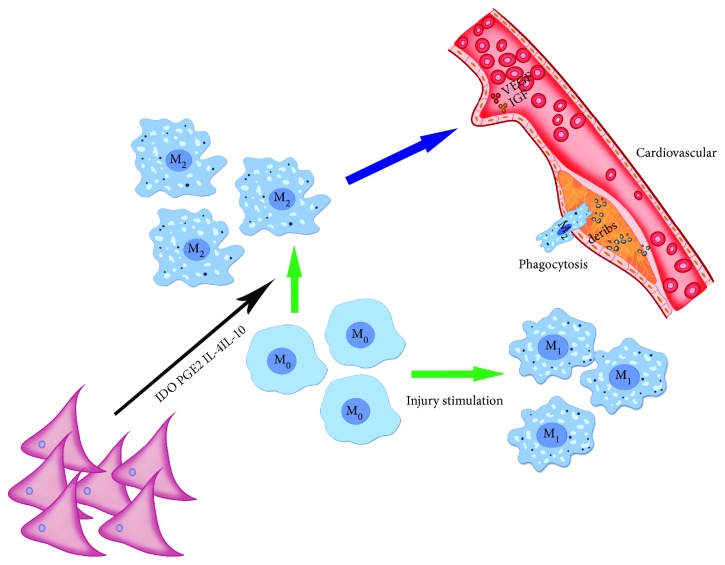
MSCs promote the differentiation of M0 to M2, which secretes VEGF, and IGF promotes recanalization of blood vessels. Green arrow: M0 differentiates into M1 and M2. Blue arrow: M2 secretes cytokines that promote recanalization of blood vessels. Black arrows: MSCs promote the differentiation of M0 to M2.

Cardiovascular disease is caused by damage to myocardial cells. Cardiomyocytes have long been considered to be highly differentiated cells and do not have the ability to regenerate following injury [101]. However, it has been shown that in the process of cardiac damage, myocardial cells and cardiac stem cells around the injured area can migrate under the promotion of inflammatory cells and quickly reenter the cell cycle, thereby promoting the recovery of cardiac function [102, 103]. Current stem cell-based therapies have provided new treatments for ischemic heart damage and heart failure; however, in the absence of nutrients and oxygen in the microenvironment, the regenerative repair of stem cells declines [104, 105]. Thus, the creation of a suitable microenvironment is also important. In the early stages of heart damage, activation of classical M1 macrophages will clear debris and produce proinflammatory cytokines such as IL-1*β*, TNF-*α*, and IFN-*γ* [106]. Different subpopulations [107, 108] were discovered in the late stage of cardiac injury, with similar phenotypes to those of anti-inflammatory M2 macrophages, showing that the presence of MSC promotes the differentiation of macrophages into M2 subtypes (Figure 4). Many molecules have been identified as being involved in this process, including IDO, PGE2, and MSC-derived IL-4 and IL-10 [7, 38, 109]. MSCs secrete TGF-*β*1 which together with PGE2 reduces macrophage-induced inflammatory factors such as IL-1*β*, IL-6, TNF-*α*, and IFN-*γ* [7, 109]. M2 macrophages promote angiogenesis by secreting anti-inflammatory and angiogenic cytokines, as well as promoting infarct healing and myocardial remodeling. M2 macrophages can also secrete growth factors, such as IGF-1 [110], to improve recovery following myocardial infarction [111]. M2 macrophages secrete vascular endothelial growth factor-A, which improves cardiac function following myocardial infarction by promoting angiogenesis. By studying the role of MSCs in an acute myocardial infarction mouse model [109], it was found that MSCs can reduce overall macrophage/monocyte counts (including M1 and M2 macrophages). However, the proportion of M2 macrophages increased significantly. Transplantation of MSCs significantly improved cardiac function and reduced myocardial fibrosis in the MSC-transplanted and nontransplanted groups following myocardial infarction [112]. In the MSC-transplanted group, capillary density increased around the infarct and M2 macrophages increased significantly at the site of transplantation. In another myocardial infarction study in mice, depletion of macrophages in the MSC-free group increased the incidence of myocardial infarction [113].

In the study of MSCs regulating the differentiation of M1 and M2 against myocardial injury, there is still no mechanism study on how MSCs regulate the regulation of inflammation into M2, thus providing a treatment for myocardial injury and fibrosis.

## 3. Application of TE Scaffold Composite MSCs

### 3.1. Immune Regulation of Cartilage Tissue Repair with MSC Seed Cells and 3D Scaffolds

The construction of cartilage TE takes into account the 3D microstructure of the selected material, as well as its biocompatibility and mechanical properties. Alternative materials, including the ECM, hydrogels, and polymers, have been used extensively in cartilage TE. For polymer scaffolds, cells should be contained within the internal structure of the polymer scaffold, such that they are retained in the body for a long period of time. Through appropriate manufacturing methods, porous scaffolds can help cells infiltrate the scaffold when implanted in the body [114, 115]. In a 3D hydrogel construct, coculture with autologous chondrocytes and MSCs may show a significantly higher rate of chondrogenesis [116]. Synthetic ECM must take into account several factors, including mechanical properties, which allow functional tissue growth and provide suitable cell-matrix interactions to stimulate tissue growth [117, 118]. Currently, the 3D behavior of specific cells (including MSCs) is considered to be different from the 2D behavior, suggesting that the cell generation environment can be imitated more closely in 3D *in vitro* culture systems compared with 2D culture [119, 120]. In the study of cartilage 3D scaffolds, scaffolds for tissue engineering (TE) are very similar to the physicochemical properties of natural extracellular matrices (ECM) and have been shown to facilitate cell attachment, proliferation, migration, and new tissue formation [121] (Table 2).

In the study of Corradetti et al., a biomimetic scaffold based on chondroitin sulfate was proposed, which can preserve the immunosuppressive potential of MSC in vitro, can respond to the immunomodulatory effects of proinflammatory cytokines, and can be immunosuppressive in the scaffold construct group. The production of molecules related to nitric oxide and prostaglandins and the expression of their inducible enzymes (iNos, PGEs, Cox-2, and TGF-*β*) are significantly increased [122]. In the Du et al. study, alginate/hyaluronic acid (Alg/HA) hydrogel scaffolds were used and bone marrow and adipose tissue-derived MSCs were induced into chondrocytes under three-dimensional conditions. MSCs before and after chondrocyte differentiation were treated with or without treatment of inflammatory conditions of IFN-*γ* and TNF-*α*, and the construct was found to have low immunogenicity and exert immunosuppressive effects on HLA-mismatched PBMC and undifferentiated MSCs [123]. Taken together, the activation of MSCs' immunoregulatory ability is related to scaffold material composition and mechanical properties. CS and HA have shown an effect in the immune regulation of MSCs. Moreover, it was found that the maintenance of the immunomodulatory ability of MSCs after differentiation was related to the microenvironment constructed by different materials, but there was no mechanism to study the immune regulation of MSCs by materials.

In the Butler et al. study, engineered cartilage with immunomodulatory properties was developed in conjunction with gene therapy and functional tissue engineering. Chondrogenesis was performed in the presence of IL-1 by inducing overexpression of an IL-1 receptor antagonist (IL-1Ra) in MSCs on the scaffold. A construct that painfully delivers a modulating anti-inflammatory cytokine enhances cartilage repair [124]. It was found that the treatment of proinflammatory factors inhibited the cartilage differentiation of MSCs to a large extent and maintained the immunoregulatory effect of MSCs, but the mechanism of tissue MSC differentiation was not explored in the article.

In the study of Zhang et al., the 3D structure affects the immunological properties of stem cells and the interaction between seed cells and the immune system of the host. Experimental results have shown that addition of the hydrogel structure helps to reduce the immune response generated following implantation of MSCs in the scaffold. Therefore, the supportive and isolating effects of 3D microstructured scaffolds (such as hydrogels prepared from higher collagen concentrations) can further reduce the immune response during transplantation, thus making them more suitable as candidates for cartilage TE [125]. In the Xingdong Zhang study, scaffold structures regulate the secretion of MSCs' immunoregulatory factors in allogeneic cartilage tissue engineering. The 3D groups (hydrogels and sponges) are more effective than under 2D monolayer culture conditions in promoting mRNA expression and protein production of soluble immune-related factors. The supernatant collected in the 3D group showed inhibition of allogeneic lymphocyte activation. The scaffold structure can regulate the secretion of MSCs. Research has allowed tissue regeneration scaffolds to control host immune rejection through immune regulation [126]. In the experiments, it was found that the 3D hydrogel was lower in expression of MHC-II on the MSCs than in the 2D culture, and the 3D scaffold produced a retraction effect under the action of MSCs, which in turn affected the immune regulation of MSCs. However, the interaction between the scaffold structure and MSCs need to be further experimentally analyzed from the mechanism.

In Ding et al.'s study, inflammation was inhibited by increasing M2 polarization of macrophages based on the engineered cartilage of BMSCs. This study indicated that the BMSC-based engineered cartilage inhibits inflammation *in vivo* through changes in the macrophage phenotype and that the tissue exhibits improved survival compared with the use of chondrocytes alone or in combination with BMSCs. BMSC-inoculated constructs improve stent-induced inflammation and promote cartilage tissue regeneration through M2 polarization of macrophages [70]. The 3D scaffold combined with MSCs promoted the recruitment and polarization of M2, but did not study the underlying mechanism of MSC-induced M2 cell recruitment and polarization.

In the study of Liu et al., the scaffold loaded with MSCs derived from human umbilical cord Wharton's jelly mesenchymal stem cells (hWJMSCs) reduced the immune response to subcutaneous implantation. hWJMSCs implanted on the back of rats did not induce an immune response in the subcutaneous environment during the observation period. The use of novel undifferentiated hWJMSCs as seed cells may be a better method for *in vivo* TE treatment of cartilage defects than differentiated hWJMSCs induced using TGF-*β* [127]. For ECM combined with MSCs, the former can promote the immune regulation of MSCs, but the xenograft of MSCs in the article is currently controversial. In the experiment, different anti-inflammatory factors were compared but lacked resistance of MSCs.

### 3.2. Immunoregulatory Effects of MSCs in Bone TE

The incorporation of MSCs into TE biomaterials is an extensively researched strategy aimed at accelerating bone formation and osteointegration during bone defect repair and regeneration. TE-related 3D delivery methods in MSC clinical application studies and the combination of porous scaffolds and MSCs have been reported for the treatment of critical-sized defects [128]. The 3D delivery of MSCs has been explored as a new strategy to improve cell delivery, functional activation, and retention in the body, to improve treatment outcomes (Table 2).

He et al.'s study was the first to investigate how macrophages in TG-gel affect bone formation in bone marrow mesenchymal stem cells (BMMSCs). It was found that macrophages encapsulated in a low-stiffness matrix played an active role in the osteogenesis of cocultured BMMSCs [129]. It was found that under 3D culture conditions, the hardness of the scaffold could have a strong influence on the polarization of macrophages. The high-hardness gel material would differentiate macrophages into M1 and prevent osteogenic differentiation of MSCs, but in the experiment the mechanism by which MSCs interact with macrophages in this process was not further explored.

Niu et al.'s study designed an injectable, transient coagulation coating material (acBSP) based on the unique macrophage affinity of glucomannan polysaccharide and can effectively promote the adhesion and activation of macrophages and mesenchymal stem cell load. Hydrogels demonstrate potent macrophage activation. The osteogenesis is achieved by activating macrophages [130]. The regulation of T cells by MSCs plays a key role in bone repair. The experiment can further explore the interaction of scaffolds with MSCs on T cells and macrophages and study the mechanism of material influence.

In a study by Arinzeh et al., autologous MSCs were loaded into hollow cylinders of hydroxyapatite-tricalcium phosphate and implanted in femoral defects in dogs. Imaging assessment, histology, and serum antibody assessment were performed at 4, 8, and 16 weeks, respectively, and no severe inflammatory reaction was found [131]. This experiment was carried out earlier to study the role of MSCs in promoting the regeneration of bone by affecting inflammatory response. It was found that allogeneic bone transplantation was consistent with autologous bone graft in the presence of MSCs, but no more molecular and cellar levels were detected.

Three-dimensional cultures of MSC/ECM complexes (C-MSCs) have been shown to repair bone damage. C-MSCs can regulate cell function *in vitro* and use the ECM as a scaffold to induce successful bone regeneration and enhance the immunoregulatory capacity of C-MSCs. MSC xenotransplantation, which exerts immunoregulatory properties by upregulating IDO activity *in vitro*, can attenuate xenogeneic reactive host immune responses and thereby induce bone regeneration in mice [31]. After pretreatment of MSCs with IFN-g, the immunogenicity of MSCs should be further tested and the relationship with bone regeneration further explored, and the mechanism of modulating T cells with MSCs needs further experimental research.

### 3.3. Immunoregulatory Effects of MSCs in Liver TE

The construction of the functional TE liver has been increasingly favored by researchers, and the emerging concept of “organizational engineering” has been proposed. With the development of TE technology, scaffolds of collagen and polymer materials have been used to evaluate their support for cell growth, liver-specific functions, and regenerative capabilities [132, 133]. The whole organ decellularization technique can largely preserve natural tissue and the macroscopic 3D structure of the liver, ensuring biocompatibility and allowing extensive cell regeneration to occur [134]. Liver ECM composition, topography, and biomechanical properties influence cell-matrix interactions. Recent advances in stent fabrication techniques for complex cells have led to the evolution of decellularized hepatic tissue matrices from a simple 2D culture to a 3D porous scaffold (Table 2).

Tian et al.'s research reveals the protective effects of MSCs and elucidates the underlying mechanisms of immune regulation in liver transplantation models. And this work provides a promising and viable option for clinical application of MSC infusion to protect liver grafts and prolong survival after transplantation [135]. The regulation of the paracrine effects of MSCs on immune cells was found in the experiment, and the role of the apoptotic process in this process was elucidated. But this immune regulation involves more other mechanisms that still require further experimental research. And the lack of relevant clinical trials of MSCs transplantation demonstrated the survival of MSCs.

In the study of Zhao et al., MSC transplantation can effectively improve liver function and reduce the number and activity of peripheral blood and liver neutrophils in acute liver failure (ALF) rats [136]. Although the immune regulation of MSCs used together with antineutrophil serum was found in the experiment, the lack of inflammatory factors involved in ALF neutrophil mediated immune regulation.

One study showed that hepatic injury could be treated with decellularized liver tissue (DLS) as scaffold-derived composite human umbilical cord-derived mesenchymal stem cells (hUC-MSCs). hUC-MSCs in 2D culture express higher levels of human leukocyte antigen-DR and IFN-*γ* compared with 3D culture and reduce the prostaglandins that inhibit lymphocyte proliferation and PGE2 secretion. The 3D-DLS system has been shown to exhibit higher immunosuppressive capacity than the *in vitro* 2D culture [137]. MSCs exhibit low immunogenicity but are enhanced after differentiation into the liver. The mechanism is still unknown. The protection of ECM scaffolds on the low immunogenicity of MSCs was pointed out, but the mechanism was not further tested.

In tissue engineering treatment of liver injury, the current method is transplant MSC-based. In order to ensure the stable existence of MSC, MSC-combined scaffold construction is one of the future development directions of liver injury tissue engineering.

### 3.4. Immune Regulation of MSCs and the Effect of MSC Immunomodulation on Myocardial Regeneration in a 3D Structure

MSCs continue to be investigated for their potential application in the restoration of myocardial function following injury. However, conventional single-layer cell cultures on the surface of 2D scaffolds do not mimic the cell-generated microenvironment well. Thus, cell cultures that have developed various 3D scaffolds further mimic the cell-producing microenvironment in which cells naturally exist and have been used to provide a platform for cell growth and transportation (Table 2). As a substrate, 3D collagen is an attractive bioengineering method for myocardial repair because collagen is a natural polymer and the primary component of the ECM of heart muscle [138, 139]. The 3D culture environment of cells also changes the biological characteristics of MSCs, including lineage differentiation [140–146], and further enhances its therapeutic efficacy [143, 147, 148]. However, the mechanism underlying this functional improvement is unknown to a large extent (Table 2).

In the study by Papalamprou et al., de-antigen scaffolds and murine MSCs were used as controls to assess MSCs to provide any additional benefit in terms of specific immune responses. Surprisingly, although mMSCs in the scaffold construct group exert immunomodulatory benefits compared to the scaffold group alone, the mMSCs vaccinated in the de-antigenic scaffolds are still immunostimulatory and can cause chronic inflammation [149]. It was found that the use of materials should retain the stem cell characteristics of MSCs as much as possible to maintain the immunomodulatory properties of MSCs to the greatest extent, but the interaction mechanism between ECM scaffolds and MSCs should be studied experimentally.

Shin et al.'s study identified a novel mechanism by which mesenchymal stromal cells (MSCs) and hydrogel scaffold constructs can reduce the recruitment of innate immune cells by locally producing adenosine. Mesenchymal stromal cells regulate excessive inflammation: implanted MSCs are found to increase the bioavailability of adenosine by the action of CD73 (ecto-50-nucleotidase). This is essential for reducing early innate immune cell infiltration and ROS formation, and mesenchymal stromal cells regulate excessive inflammation [150]. In the experiment, because of the small sample size, although the authors made a comparison between the MSCs and the carrier scaffold, but did not detect the difference between the hydrogel scaffold group and the blank group, further experimental proof is needed. And because of the importance of CD73, the necessity of further research on the implantation of MSCs is proposed.

The potential for cardiac repair has been shown to be limited in standard 2D cultures, and fiber characteristics develop as culture time increases. Three-dimensional collagen scaffolds can enhance the production of trophic factors, modify their immunomodulatory and fibrogenic phenotypes, and promote the cardioprotective effects of MSCs. MSCs have been shown to maintain an antiapoptotic effect and enhance the expression of cardiac trophic factors in 3D collagen scaffolds. Understanding the mechanism of MSC-mediated tissue repair will help to further improve the therapeutic efficacy of MSCs [151]. The effect of the scaffold on MSC is attributed to the biophysical properties of 3D, and the hardness of the ECM scaffold is a key influencing factor. However, biochemical effects were not excluded in this experiment and may both work at the same time; further experimental proof is needed.

MSC-seeded plasma-coated PCL grafts were beneficial for cardiac function in a rodent model of myocardial infarction. By examining the recruitment of macrophages, significantly fewer CD68^+^ macrophages were found in the MSC composite scaffold group than in the control group, indicating significant anti-inflammatory effects [152]. The experimental results show that the chemical and structural characteristics of the scaffold can regulate the occurrence of immune response, and the early inflammation response of the scaffold during the delivery of MSC is regulated by MSC, which may play an important role in the late stage of inflammation. Further experiment is needed to investigate the balance of inflammation between myocardial repair and fibrosis.

## 4. Perspectives and Conclusions

The rapid development of regenerative medicine has made it possible to repair damaged tissues with the help of stem cells. With increased research on stem cell repair, it is clear that the immune system plays a key role in stem cell repair-mediated tissue regeneration. According to regulation of the immune system by stem cells, paracrine production by the stimulation of immune cells in damaged tissue may be another promising therapeutic strategy. The use of stem cells and immune cells to regulate inflammation in areas of tissue damage can be used to reduce disease progression in cases with chronic tissue injury. In a series of tissue repairs, it is clear that the immune system plays an important role in tissue regeneration and repair. However, at the same time, the effects of stem cell differentiation on immunoregulatory activity still require further experimental investigation.

The role of stem cells in regulating the immune microenvironment is affected by the addition of TE technology. It is mentioned that MSCs under the influence of 3D scaffolds increase the secretion of anti-inflammatory cytokines and reduce the infiltration of inflammatory cells. The choice of 3D scaffolds in different tissues (cartilage, bone, liver, and myocardium) is particularly important in the scaffold structure as well as material properties and the preconstruction inflammatory microenvironment. In this review, 3D scaffolds of different organs are exemplified in the immune regulation of MSC. MSCs in the treatment of cartilage damage, tissue engineering scaffolds of 3D structure of different materials (hydrogel, cell-based ECM) affect MSC immune regulation and promote cartilage tissue repair. In the treatment of myocardial injury, 3D scaffold (collagen, polymer material) composite MSC for the treatment of anti-inflammatory fibrosis in myocardial injury. In the treatment of liver injury, 3D decellularization scaffold liver scaffold composite stem cells affect the secretion of inflammatory factors. In the treatment of bone injury, ECM and polymer material combined with MSCs to regulate the immune microenvironment promotes bone tissue regeneration.

Multidisciplinary research will be imperative to this end, and TE technology provides a vehicle for the transport of cells and paracrine factors. In some situations, TE can meet the mechanical requirements. Combined with the organizational engineering technology developed by medical personnel, TE now provides new ideas for organizational repair. However, the best means of constructing a reasonable immune microenvironment remains an important issue faced by TE technology. Combined with stem cell treatment of tissue defects, 3D scaffolds can provide a reasonable carrier for stem cells and enhance the immunosuppressive effect of MSCs. However, at the same time, inhomogeneous oxygen and nutrient distribution in the 3D spatial structure also impact on stem cells. The stem cell microenvironment will be an important factor in stem cell TE.

To achieve greater clinical efficacy in the future, we should focus on the construction of the immune microenvironment of damaged tissues and the use of TE in the construction of a suitable microenvironment. In terms of regenerative therapy, multidisciplinary cooperation and a greater understanding of the microenvironment will be important for future developments.

## Figures and Tables

**Table 1:** 

Material	Scaffold structure	Stem cell source	Stem cell pretreatment	Function	Reference
MSC	3D spheroid	Human bone marrow-derived	Interleukin- (IL-) 1	The 3D MSC construct was reduced in LPS-induced TNF-a secretion and decreased IL-6 secretion	[58]
PLA/chitosan	Cylinders	Human bone marrow-derived	no	MSC interaction with macrophage within 3D scaffolds hampers fibroblast recruitment	[60]
MSC	3D spheroid	Human bone marrow-derived	IFN-g	MSCs express high levels of proliferating genes, lower levels of inflammation, apoptosis, and senescence genes in 3D	[59]
MSC	3D spheroid	Human adipose-derived	no	Increased angiogenic cytokine levels and immunosuppression against apoptosis in MSC spheroids	[57]
MSC	ALN bioreactor system	Rat bone marrow-derived	no	High function efficacy of MSC in the ALN-reactor system than the 2D culture	[61]

**Table 2:** 

Organ	MSC source	MSC pretreatment	Scaffold type	Immunomodulatory	Reference
Cartilage	Rabbit bone marrow-derive	IFN-g	Hydrogel scaffold	The hydrogel structure helps to reduce the immune response of MSCs after vaccination, even in the presence of inflammation cytokines.	[125]
	Human bone marrow-derived	IFN-g TNF-a	Alginate/hydrogel scaffold	MSCs under 3D hydrogel have low immunogenicity and can exert an immunosuppressive effect on HLA-mismatched PBMCs. And it has an inhibitory effect in NK cell-mediated cytolysis.	[123]
	Human bone marrow-derived	Overexpression of IL-1 receptor antagonist in MSCs induced by lentivirus	Woven PCL scaffold	Enhancement of collagen/GAG production in scaffolds expressing IL-1Ra under inflammatory conditions MMP was reduced in the construct compared to the untreated group, and the level of PGE2 was elevated.	[124]
	Rat bone marrow-derived	No	Hydrogel-sponge concentration	The production of NO, PGE2, HGF, and IDO increased gradually in 2D culture, and the immunoregulatory factor secreted by MSC in the 3D group reduced the activation ability of allogeneic lymphocytes.	[126]
	Pig bone marrow-derived	No	Cylindrical unwoven PGA fiber	The cytokines IL-10 and TGF-b were increased in the construction group, and the ability of FBGC recruitment was decreased.	[70]
	Human umbilical cord-derived	No	Decellularized pig ECM scaffold	Molecular IDO, PEG2, TGF-b1, IL-10, VEGF, and HGF increased in the scaffold concentration group.	[127]
	Rat bone marrow-derived	TNF-a	Freeze-dried collagen scaffolds	The scaffold construct group exhibited an immunosuppressive potential with a significant increase in iNos. And an upward trend was also observed for Cox and TGF-b.	[122]

Bone	mice bone marrow-derived	No	Transglutaminase glutathionase-crosslinked gelatin (TG-gel)	Cytokines and gene profiles of TNF-a and IL-10 in the scaffold construct showed elevated cincentractions in the test group.	[129]
	Human bone marrow-derived	No	3D instantaneously solidifying material (acBSP)	The scaffold construct synergizes with macrophage to promote cytokine expression of IL-11, IL-17, IL-4, and IL-6 and low expression of IL-1b and TNF-a.	[130]
	Human bone marrow-derived	No	MSC loaded on hydroxyapatite-tricalcium phosphate	After implantation of the scaffold construct, histologically, no lymphocytic infiltration occurred. And new bone was formed throughout the implant.	[131]
	Human bone marrow-derived	IFN-g	ECM	The scaffold construct can induce bone regeneration and inhibit xenografting of mouse T cells in the transplanted area.	[31]

liver	Mice bone marrow-derived	No	MSC transplantation	In the experiment group, TNF-a, IFN-g, IL-2, IL-17, IL-1b, and MPO secretion was decreased, and IL-10 was reversed. Expression of CXCL1, CCL2, CCL4, CCL7, and CXCL10 was inhibited.	[135]
	Rat bone marrow-derived	No	MSC transplantation	The expression of TNF-a, IL-1b, CXCL1, and CXCL2 was decreased, and the expression of the anti-inflammatory cytokine IL-10 was increased.	[136]
	Human umbilical cord-derived	No	3D spheroid	PGE2 secreted by the 3D group was significantly increased, and IFN-g was decreased.	[137]

Heart	Mice bone marrow-derived	No	Decellularized ECM	MSC vaccination results in positive immunomodulatory effect but a persistent chronic inflammatory response.	[149]
	Rat bone marrow-derived	No	3D hydrogel	Compared with the control group, the scaffold construct played a role in inhibiting leukocyte and promoting repair in the late stage of inflammation.	[150]
	Human bone marrow-derived	Simulated inflammatory environment	3D collagen scaffold	The immunosuppressive function of MSCs is retained in the 3D scaffold and promotes the activation of M2 macrophage. Single-layer cocultures with IL-10 levels lower than MSCs	[151]
	Rat bone marrow-derived	No	PCL	For the infiltration of CD68(+) macrophage in the absence of the scaffold construct, and the control group had a higher number of CD68(+)	[152]
